# National and State Trends in Anxiety and Depression Severity Scores Among Adults During the COVID-19 Pandemic — United States, 2020–2021

**DOI:** 10.15585/mmwr.mm7040e3

**Published:** 2021-10-08

**Authors:** Haomiao Jia, Rebecca J. Guerin, John P. Barile, Andrea H. Okun, Lela McKnight-Eily, Stephen J. Blumberg, Rashid Njai, William W. Thompson

**Affiliations:** ^1^Department of Biostatistics, Mailman School of Public Health and School of Nursing, Columbia University, New York, New York; ^2^Division of Science Integration, National Institute for Occupational Safety and Health, CDC; ^3^Department of Psychology, University of Hawaii at Manoa, Honolulu, Hawaii; ^4^Division of HIV/AIDS Prevention, National Center for HIV/AIDS, Viral Hepatitis, STD, and TB Prevention, CDC; ^5^Division of Health Interview Statistics, National Center for Health Statistics, CDC; ^6^Office of Minority Health, CDC; ^7^CDC COVID-19 Response Team, CDC; ^8^Division of Viral Hepatitis, National Center for HIV/AIDS, Viral Hepatitis, STD, and TB Prevention, CDC

Recent studies indicate an increase in the percentage of adults who reported clinically relevant symptoms of anxiety and depression during the COVID-19 pandemic (*1*–*3*). For example, based on U.S. Census Bureau Household Pulse Survey (HPS) data, CDC reported significant increases in symptoms of anxiety and depressive disorders among adults aged ≥18 years during August 19, 2020–February 1, 2021, with the largest increases among adults aged 18–29 years and among those with less than a high school education (*1*). To assess more recent national trends, as well as state-specific trends, CDC used HPS data (*4*) to assess trends in reported anxiety and depression among U.S. adults in all 50 states and the District of Columbia (DC) during August 19, 2020–June 7, 2021 (*1*). Nationally, the average anxiety severity score increased 13% from August 19–31, 2020, to December 9–21, 2020 (average percent change [APC] per survey wave = 1.5%) and then decreased 26.8% from December 9–21, 2020, to May 26–June 7, 2021 (APC = –3.1%). The average depression severity score increased 14.8% from August 19–31, 2020, to December 9–21, 2020 (APC = 1.7%) and then decreased 24.8% from December 9–21, 2020, to May 26–June 7, 2021 (APC = –2.8%). State-specific trends were generally similar to national trends, with both anxiety and depression scores for most states peaking during the December 9–21, 2020, or January 6–18, 2021, survey waves. Across the entire study period, the frequency of anxiety and depression symptoms was positively correlated with the average number of daily COVID-19 cases. Mental health services and resources, including telehealth behavioral services, are critical during the COVID-19 pandemic.

Data were obtained from HPS (*4*), a biweekly, online survey. The survey, developed with assistance from CDC and other federal agencies to assess the social and economic impacts of the COVID-19 pandemic on U.S. households, began on April 23, 2020. Samples for HPS are drawn from an extract of the U.S. Census Bureau’s master address file that includes email and mobile telephone numbers of approximately 117 million U.S. housing units. Survey data include sample weights to be used in analyses to generate results representative of U.S. adults aged ≥18 years based on age, sex, race/ethnicity, and educational attainment; the data from this experimental product were designed to produce estimates at state and national levels.* This analysis examined data for adults aged ≥18 years collected from the 19 biweekly surveys (waves) conducted during August 19, 2020–June 7, 2021 (waves 13–31), with breaks during December 22, 2020–January 5, 2021, because of expected decreases in survey response rates during holiday seasons, and during March 30–April 13, 2021, when HPS transitioned to a new survey cycle (Supplementary Table 1, https://stacks.cdc.gov/view/cdc/110122). The total sample size was 1,526,154 for all 19 waves, ranging from 58,729 (wave 18) to 110,019 (wave 14). Overall survey response rates ranged from 5.3% to 10.3% among the 19 waves examined.

Frequency of experiencing anxiety and depressive symptoms was assessed using the four-item Patient Health Questionnaire (PHQ-4),^†^ which includes the two-item Generalized Anxiety Disorder (GAD-2) scale and the two-item PHQ-2, which assesses symptoms of depression (*5*). For each survey response, answers were assigned a numerical value: not at all = 0, several days = 1, more than one half of the days = 2, and nearly every day = 3. The anxiety severity score was calculated by summing the two GAD-2 responses, and the depression severity score was calculated by summing the two PHQ-2 responses. The sum of both severity scores could range from 0 to 6.

Weighted mean anxiety and depression severity scores for the United States were calculated for each wave. For state estimates, weighted age-standardized mean scores were calculated using direct standardization. State-specific trends were modeled using linear mixed models with cubic splines for sampling waves (*6*) to obtain smoothed age-adjusted estimates for each wave. The age-standardized state-level average anxiety and depression severity scores for the 50 states and DC are presented for the first two waves (August 19–September 24, 2020), the peak period in anxiety and depression severity scores nationally (December 9, 2020–January 18, 2021), and the last two waves (May 12–June 7, 2021). APCs for anxiety and depression severity scores per wave were calculated, and bootstrapping was used to obtain 95% confidence intervals (CIs) for APCs^§^ (*7*). A change in the mean severity score indicates a change in the frequency of symptoms. To allow relative comparisons of HPS estimates with those from a nonpandemic period, weighted means and CIs were calculated for anxiety and depression severity scores using data from the 2019 National Health Interview Survey (NHIS), with data from 31,997 adults aged ≥18 years.^¶^ Daily numbers of COVID-19 cases were obtained from the CDC COVID Data Tracker and were averaged across the same periods that the HPS anxiety and depression symptom data were collected. Average counts of daily cases were compared with the symptom data using Pearson correlations. This activity was reviewed by CDC and was conducted consistent with applicable federal law and CDC policy.**

Nationally, the average anxiety severity scores increased from 2.0 during August 19–31, 2020, to 2.3 during December 9–21, 2020 (APC = 1.5% per wave), reflecting a 13.0% increase in symptoms (Figure) (Supplementary Table 2, https://stacks.cdc.gov/view/cdc/110123). During this same period, the average depression severity score increased from 1.6 to 2.0, reflecting a 14.8% increase. From December 9–21, 2020, to May 26–June 7, 2021, the average anxiety severity score decreased to 1.7 (APC = −3.1% per wave), reflecting a 26.8% decrease; during this same period, the average depression severity score decreased to 1.4 (APC = −2.8% per wave), reflecting a 24.8% decrease.

**FIGURE:**
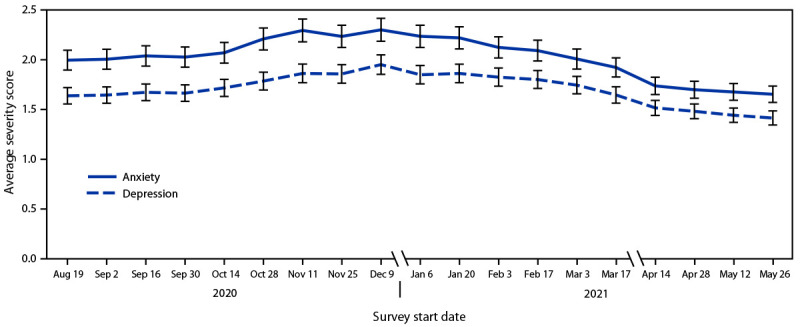
Trends in average anxiety and depression severity scores* among adults, by survey start date — Household Pulse Survey, United States, August 19, 2020–June 7, 2021^†^ * 95% confidence intervals indicated by error bars. ^^†^^ Data for adults aged ≥18 years were collected from 19 biweekly surveys (waves) conducted during August 19, 2020–June 7, 2021 (waves 13–31), with breaks during December 22, 2020–January 5, 2021, and March 30–April 13, 2021.

Analyses of 2019 NHIS data indicate that the weighted average anxiety and depression severity scores among adults aged ≥18 years were 0.63 and 0.51, respectively. Quarterly weighted average anxiety and depression severity scores ranged from 0.61 to 0.65 and from 0.50 to 0.52, respectively; variation in these quarterly scores was substantially less than that in HPS during similar months.

In most states, the average anxiety and depression severity scores increased from August–September 2020 to December 2020–January 2021 (Table 1) (Supplementary Figure, https://stacks.cdc.gov/view/cdc/110121). By May–June 2021, anxiety and depression severity scores were similar to or lower than those during August–September 2020. During August–December 2020 and January–June 2021, state-level trends in anxiety and depression severity scores were similar to national trends, with scores for most states peaking during December 9–21, 2020, or January 6–18, 2021. States with larger increases in severity scores during August–December 2020 also tended to have larger decreases during January–June 2021 (Table 2). Mississippi, Oklahoma, and South Carolina had the largest percentage increases in anxiety scores during August–December 2020, whereas Minnesota, Mississippi, and South Carolina had the largest percentage increases in depression scores; Florida and New York had the smallest percentage increases in depression and anxiety scores, respectively. During January–June 2021, Minnesota, Rhode Island, and Utah had the largest percentage decreases in anxiety scores; Idaho, Michigan, and Wisconsin had the largest percentage decreases for depression severity scores, whereas New York had the smallest decrease in both anxiety and depression scores.

**TABLE 1:** Average anxiety and depression severity scores, by state/area — Household Pulse Survey, United States, August–September 2020, December 2020–January 2021, and May–June 2021

State/Area	Mean severity score (95% CI)					
	Anxiety			Depression		
	Aug 19–Sep 14, 2020	Dec 9, 2020–Jan 18, 2021	May 12–Jun 7, 2021	Aug 19–Sep 14, 2020	Dec 9, 2020–Jan 18, 2021	May 12–Jun 7, 2021
Alabama	2.02 (1.90–2.15)	2.38 (2.19–2.57)	1.75 (1.49–2.00)	1.73 (1.61–1.84)	1.99 (1.81–2.16)	1.48 (1.26–1.71)
Alaska	1.84 (1.73–1.95)	2.24 (2.11–2.36)	1.51 (1.32–1.70)	1.54 (1.43–1.64)	1.89 (1.77–2.02)	1.29 (1.12–1.46)
Arizona	2.05 (1.95–2.15)	2.16 (2.05–2.27)	1.82 (1.62–2.01)	1.77 (1.68–1.87)	1.92 (1.80–2.04)	1.50 (1.34–1.67)
Arkansas	2.06 (1.94–2.19)	2.35 (2.16–2.54)	1.73 (1.46–1.99)	1.74 (1.63–1.85)	1.92 (1.75–2.09)	1.51 (1.29–1.73)
California	2.12 (2.05–2.20)	2.45 (2.35–2.55)	1.73 (1.61–1.86)	1.80 (1.72–1.87)	2.06 (1.96–2.16)	1.51 (1.39–1.62)
Colorado	2.04 (1.94–2.14)	2.30 (2.18–2.42)	1.78 (1.59–1.97)	1.64 (1.56–1.72)	1.90 (1.78–2.01)	1.41 (1.24–1.58)
Connecticut	2.09 (1.97–2.20)	2.39 (2.24–2.55)	1.53 (1.31–1.74)	1.61 (1.51–1.71)	1.97 (1.83–2.11)	1.21 (1.02–1.40)
Delaware	1.84 (1.69–1.99)	2.26 (2.08–2.44)	1.74 (1.45–2.03)	1.58 (1.43–1.72)	1.90 (1.73–2.07)	1.43 (1.17–1.70)
District of Columbia	2.05 (1.85–2.24)	2.10 (1.91–2.28)	1.59 (1.38–1.79)	1.59 (1.40–1.78)	1.58 (1.42–1.74)	1.35 (1.15–1.54)
Florida	2.10 (2.01–2.20)	2.31 (2.17–2.45)	1.67 (1.48–1.85)	1.73 (1.64–1.81)	1.85 (1.72–1.97)	1.36 (1.19–1.52)
Georgia	1.96 (1.86–2.06)	2.32 (2.17–2.47)	1.78 (1.57–1.99)	1.63 (1.53–1.72)	1.93 (1.78–2.07)	1.50 (1.32–1.68)
Hawaii	2.17 (2.01–2.34)	2.02 (1.81–2.23)	1.81 (1.54–2.07)	1.80 (1.65–1.94)	1.66 (1.46–1.85)	1.47 (1.22–1.71)
Idaho	1.91 (1.80–2.02)	2.18 (2.04–2.33)	1.59 (1.33–1.84)	1.54 (1.44–1.64)	1.88 (1.74–2.02)	1.23 (1.04–1.43)
Illinois	2.04 (1.95–2.14)	2.27 (2.14–2.40)	1.64 (1.45–1.83)	1.62 (1.54–1.71)	1.79 (1.68–1.90)	1.43 (1.24–1.62)
Indiana	1.95 (1.85–2.05)	2.11 (1.98–2.24)	1.67 (1.50–1.85)	1.56 (1.47–1.65)	1.75 (1.62–1.87)	1.45 (1.28–1.62)
Iowa	1.82 (1.72–1.92)	2.14 (1.98–2.31)	1.57 (1.36–1.78)	1.50 (1.40–1.60)	1.87 (1.72–2.02)	1.38 (1.18–1.58)
Kansas	1.90 (1.81–2.00)	2.18 (2.04–2.32)	1.53 (1.37–1.70)	1.52 (1.43–1.61)	1.86 (1.71–2.01)	1.30 (1.15–1.45)
Kentucky	2.13 (2.01–2.25)	2.36 (2.18–2.54)	1.64 (1.39–1.90)	1.81 (1.69–1.92)	2.00 (1.84–2.16)	1.57 (1.33–1.81)
Louisiana	2.25 (2.10–2.39)	2.49 (2.28–2.71)	1.86 (1.61–2.11)	1.83 (1.70–1.96)	2.11 (1.91–2.31)	1.56 (1.33–1.79)
Maine	2.14 (2.00–2.28)	2.28 (2.06–2.49)	1.54 (1.28–1.80)	1.63 (1.50–1.76)	1.81 (1.62–2.00)	1.39 (1.15–1.62)
Maryland	1.92 (1.83–2.02)	2.21 (2.08–2.34)	1.56 (1.39–1.72)	1.56 (1.47–1.65)	1.84 (1.71–1.97)	1.25 (1.12–1.37)
Massachusetts	1.98 (1.88–2.07)	2.27 (2.14–2.40)	1.62 (1.47–1.77)	1.52 (1.44–1.60)	1.79 (1.67–1.91)	1.36 (1.22–1.50)
Michigan	2.01 (1.91–2.11)	2.42 (2.29–2.55)	1.49 (1.33–1.66)	1.62 (1.53–1.71)	2.07 (1.93–2.20)	1.34 (1.18–1.50)
Minnesota	1.79 (1.71–1.87)	2.04 (1.92–2.17)	1.51 (1.35–1.67)	1.38 (1.30–1.45)	1.69 (1.58–1.80)	1.29 (1.13–1.45)
Mississippi	1.98 (1.85–2.11)	2.47 (2.26–2.68)	1.96 (1.63–2.30)	1.71 (1.59–1.82)	2.20 (2.01–2.40)	1.74 (1.33–2.15)
Missouri	1.90 (1.80–2.00)	2.28 (2.13–2.43)	1.88 (1.65–2.12)	1.58 (1.48–1.68)	1.93 (1.76–2.10)	1.63 (1.42–1.85)
Montana	1.89 (1.75–2.02)	2.34 (2.16–2.52)	1.31 (1.08–1.55)	1.46 (1.34–1.58)	1.88 (1.71–2.05)	1.21 (0.97–1.45)
Nebraska	1.71 (1.61–1.81)	1.95 (1.81–2.09)	1.29 (1.12–1.47)	1.45 (1.36–1.55)	1.74 (1.60–1.88)	1.10 (0.93–1.27)
Nevada	2.15 (2.03–2.27)	2.40 (2.24–2.55)	1.88 (1.64–2.13)	1.78 (1.66–1.90)	2.10 (1.96–2.24)	1.66 (1.42–1.90)
New Hampshire	1.97 (1.84–2.09)	2.10 (1.93–2.27)	1.75 (1.50–2.00)	1.52 (1.40–1.65)	1.67 (1.49–1.85)	1.60 (1.37–1.84)
New Jersey	2.07 (1.96–2.17)	2.27 (2.13–2.41)	1.73 (1.53–1.93)	1.61 (1.52–1.70)	1.87 (1.74–2.01)	1.46 (1.23–1.69)
New Mexico	2.26 (2.13–2.39)	2.39 (2.23–2.55)	1.80 (1.57–2.04)	1.86 (1.74–1.99)	2.07 (1.92–2.23)	1.63 (1.40–1.86)
New York	2.07 (1.96–2.17)	2.22 (2.08–2.36)	1.63 (1.41–1.84)	1.65 (1.56–1.75)	1.82 (1.70–1.95)	1.49 (1.29–1.69)
North Carolina	1.94 (1.83–2.05)	2.27 (2.12–2.42)	1.79 (1.55–2.04)	1.52 (1.42–1.63)	1.87 (1.73–2.01)	1.56 (1.35–1.77)
North Dakota	1.55 (1.42–1.68)	1.94 (1.72–2.16)	1.46 (1.09–1.84)	1.21 (1.08–1.34)	1.56 (1.39–1.74)	1.37 (0.97–1.77)
Ohio	2.02 (1.91–2.14)	2.29 (2.12–2.46)	1.64 (1.43–1.84)	1.75 (1.63–1.86)	2.11 (1.95–2.27)	1.48 (1.27–1.69)
Oklahoma	2.10 (1.98–2.22)	2.49 (2.34–2.64)	1.76 (1.54–1.99)	1.72 (1.61–1.83)	2.09 (1.95–2.23)	1.56 (1.36–1.76)
Oregon	2.25 (2.17–2.34)	2.50 (2.39–2.61)	1.85 (1.69–2.00)	1.78 (1.70–1.87)	2.12 (2.01–2.23)	1.57 (1.43–1.72)
Pennsylvania	1.99 (1.89–2.08)	2.21 (2.07–2.34)	1.73 (1.57–1.90)	1.62 (1.53–1.71)	1.95 (1.82–2.09)	1.38 (1.22–1.53)
Rhode Island	1.99 (1.86–2.13)	2.32 (2.10–2.54)	1.62 (1.26–1.98)	1.59 (1.46–1.73)	1.85 (1.67–2.03)	1.40 (1.05–1.75)
South Carolina	1.75 (1.63–1.87)	2.23 (2.07–2.40)	1.72 (1.46–1.99)	1.44 (1.33–1.55)	1.83 (1.67–1.99)	1.50 (1.27–1.73)
South Dakota	1.57 (1.46–1.68)	1.90 (1.70–2.10)	1.51 (1.23–1.79)	1.30 (1.19–1.41)	1.60 (1.41–1.80)	1.42 (1.09–1.74)
Tennessee	1.99 (1.88–2.10)	2.25 (2.10–2.40)	1.61 (1.42–1.80)	1.67 (1.57–1.78)	1.95 (1.81–2.09)	1.38 (1.20–1.57)
Texas	1.97 (1.89–2.05)	2.25 (2.14–2.36)	1.79 (1.62–1.96)	1.67 (1.60–1.75)	1.92 (1.82–2.02)	1.54 (1.37–1.70)
Utah	1.82 (1.73–1.91)	2.14 (2.04–2.24)	1.53 (1.39–1.68)	1.48 (1.40–1.57)	1.80 (1.70–1.90)	1.31 (1.18–1.45)
Vermont	1.97 (1.82–2.12)	2.23 (2.03–2.44)	1.57 (1.27–1.87)	1.55 (1.41–1.69)	1.73 (1.55–1.91)	1.41 (1.06–1.76)
Virginia	1.94 (1.83–2.04)	2.26 (2.13–2.40)	1.67 (1.48–1.85)	1.60 (1.50–1.70)	1.88 (1.75–2.02)	1.49 (1.30–1.67)
Washington	2.02 (1.95–2.09)	2.34 (2.24–2.44)	1.78 (1.65–1.91)	1.60 (1.53–1.67)	1.97 (1.87–2.06)	1.54 (1.40–1.67)
West Virginia	2.02 (1.87–2.17)	2.58 (2.38–2.77)	1.77 (1.48–2.05)	1.67 (1.53–1.82)	2.33 (2.14–2.52)	1.51 (1.28–1.74)
Wisconsin	1.79 (1.69–1.89)	2.11 (1.97–2.25)	1.46 (1.26–1.65)	1.46 (1.36–1.55)	1.73 (1.60–1.86)	1.12 (0.96–1.28)
Wyoming	1.94 (1.80–2.07)	2.19 (1.94–2.45)	1.32 (1.04–1.60)	1.50 (1.37–1.63)	1.72 (1.51–1.92)	1.23 (1.01–1.44)

**Abbreviation:** CI = confidence interval.

**TABLE 2:** National and state average percent change in anxiety and depression severity scores, by state/area — Household Pulse Survey, United States, August 2020–June 2021

State/Area	Anxiety severity score			Depression severity score		
	Average % change* (95% CI)		Peak survey wave	Average % change* (95% CI)		Peak survey wave
	Aug–Dec 2020	Jan–Jun 2021		Aug–Dec 2020	Jan–Jun 2021	
**U.S. total**	**1.5 (1.3 to 1.7)**	**–3.1 (–3.3 to –2.9)**	**21**	**1.7 (1.5 to 2.0)**	**–2.8 (–3.0 to –2.6)**	**22**
Alabama	1.7 (0.8 to 2.6)	–2.8 (–3.7 to –2.0)	22	1.7 (0.7 to 2.7)	–2.5 (–3.4 to –1.5)	22
Alaska	1.9 (1.4 to 2.5)	–3.3 (–3.9 to –2.7)	21	2.2 (1.6 to 2.8)	–3.0 (–3.7 to –2.3)	22
Arizona	1.2 (0.5 to 1.8)	–2.6 (–3.3 to –1.9)	20	1.1 (0.3 to 1.8)	–2.6 (–3.3 to –1.9)	22
Arkansas	1.8 (1.0 to 2.7)	–2.7 (–3.6 to –1.8)	21	1.7 (0.8 to 2.6)	–2.4 (–3.3 to –1.4)	22
California	1.5 (0.9 to 2.1)	–3.2 (–3.7 to –2.7)	21	1.5 (0.8 to 2.2)	–2.6 (–3.2 to –2.0)	22
Colorado	1.6 (0.9 to 2.2)	–3.0 (–3.6 to –2.3)	20	1.6 (0.9 to 2.3)	–2.6 (–3.3 to –1.8)	21
Connecticut	1.7 (1.0 to 2.4)	–3.3 (–4.0 to –2.6)	21	2.3 (1.4 to 3.1)	–3.3 (–4.0 to –2.5)	22
Delaware	1.8 (1.1 to 2.5)	–3.0 (–3.8 to –2.3)	20	2.0 (1.2 to 2.9)	–2.8 (–3.7 to –1.9)	21
District of Columbia	1.4 (0.6 to 2.3)	–2.9 (–3.6 to –2.2)	21	1.6 (0.5 to 2.6)	–2.5 (–3.3 to –1.6)	22
Florida	1.1 (0.3 to 1.8)	–2.8 (–3.6 to –2.1)	20	0.9 (0.1 to 1.7)	–2.5 (–3.3 to –1.6)	19
Georgia	1.9 (1.1 to 2.8)	–2.7 (–3.5 to –1.9)	21	2.3 (1.4 to 3.2)	–2.7 (–3.6 to –1.8)	22
Hawaii	0.9 (0.0 to 1.8)	–2.9 (–3.7 to –2.1)	20	1.0 (0.0 to 2.1)	–2.7 (–3.6 to –1.7)	20
Idaho	1.7 (1.0 to 2.4)	–3.3 (–4.0 to –2.6)	21	2.3 (1.5 to 3.1)	–3.4 (–4.2 to –2.7)	22
Illinois	1.3 (0.6 to 2.0)	–3.0 (–3.6 to –2.3)	21	1.8 (1.0 to 2.6)	–2.7 (–3.6 to –1.9)	22
Indiana	1.5 (0.8 to 2.2)	–3.0 (–3.7 to –2.2)	21	2.0 (1.2 to 2.8)	–2.8 (–3.5 to –2.0)	21
Iowa	1.9 (1.1 to 2.7)	–3.2 (–3.9 to –2.5)	21	2.2 (1.3 to 3.1)	–3.0 (–3.8 to –2.2)	22
Kansas	1.6 (0.9 to 2.3)	–3.4 (–4.1 to –2.7)	20	2.2 (1.4 to 3.0)	–3.2 (–4.0 to –2.4)	21
Kentucky	1.2 (0.4 to 2.1)	–3.0 (–3.8 to –2.2)	21	1.2 (0.3 to 2.1)	–2.5 (–3.4 to –1.7)	22
Louisiana	1.5 (0.6 to 2.3)	–2.9 (–3.8 to –1.9)	22	2.0 (1.0 to 3.0)	–2.7 (–3.7 to –1.7)	22
Maine	1.4 (0.6 to 2.1)	–3.4 (–4.1 to –2.7)	21	2.0 (1.1 to 2.9)	–3.2 (–4.1 to –2.4)	22
Maryland	1.8 (1.1 to 2.5)	–3.3 (–4.0 to –2.7)	21	2.1 (1.3 to 2.9)	–3.2 (–4.0 to –2.5)	22
Massachusetts	1.6 (0.9 to 2.3)	–3.1 (–3.7 to –2.6)	20	2.0 (1.3 to 2.8)	–2.9 (–3.6 to –2.2)	21
Michigan	1.7 (0.9 to 2.4)	–3.5 (–4.1 to –2.8)	22	2.3 (1.5 to 3.1)	–3.5 (–4.2 to –2.8)	22
Minnesota	2.0 (1.4 to 2.7)	–3.5 (–4.1 to –2.8)	20	2.6 (1.8 to 3.4)	–3.1 (–3.9 to –2.3)	21
Mississippi	2.2 (1.3 to 3.0)	–3.4 (–4.3 to –2.5)	22	2.5 (1.5 to 3.5)	–3.3 (–4.2 to –2.3)	22
Missouri	1.9 (1.1 to 2.7)	–2.8 (–3.5 to –2.0)	21	2.0 (1.1 to 3.0)	–2.7 (–3.5 to –1.8)	22
Montana	1.7 (0.9 to 2.4)	–3.5 (–4.3 to –2.6)	20	2.2 (1.3 to 3.0)	–3.2 (–4.1 to –2.4)	21
Nebraska	1.8 (1.1 to 2.5)	–3.4 (–4.0 to –2.7)	20	2.2 (1.4 to 3.0)	–3.3 (–4.0 to –2.5)	22
Nevada	1.7 (0.9 to 2.4)	–3.4 (–4.2 to –2.7)	20	2.0 (1.1 to 2.8)	–3.3 (–4.1 to –2.5)	21
New Hampshire	1.7 (1.0 to 2.3)	–3.1 (–3.8 to –2.3)	21	2.1 (1.2 to 2.9)	–2.5 (–3.4 to –1.7)	22
New Jersey	1.3 (0.6 to 2.0)	–3.0 (–3.7 to –2.3)	21	1.7 (0.9 to 2.6)	–2.6 (–3.4 to –1.8)	22
New Mexico	1.4 (0.7 to 2.1)	–3.1 (–3.8 to –2.5)	20	1.7 (0.9 to 2.5)	–2.9 (–3.6 to –2.2)	21
New York	0.8 (0.1 to 1.6)	–2.4 (–3.2 to –1.6)	22	1.1 (0.2 to 2.0)	–1.9 (–2.8 to –0.9)	23
North Carolina	2.0 (1.2 to 2.9)	–2.7 (–3.5 to –1.9)	22	2.3 (1.3 to 3.3)	–2.5 (–3.4 to –1.6)	22
North Dakota	1.9 (1.1 to 2.7)	–3.2 (–4.1 to –2.4)	21	2.2 (1.3 to 3.1)	–2.9 (–3.9 to –1.9)	22
Ohio	1.5 (0.7 to 2.4)	–3.3 (–4.1 to –2.5)	21	1.8 (0.8 to 2.8)	–2.8 (–3.7 to –1.9)	22
Oklahoma	2.2 (1.4 to 2.9)	–3.2 (–4.1 to –2.4)	21	2.3 (1.4 to 3.2)	–2.7 (–3.6 to –1.9)	22
Oregon	1.0 (0.5 to 1.6)	–3.0 (–3.5 to –2.5)	21	1.8 (1.1 to 2.4)	–3.0 (–3.6 to –2.4)	22
Pennsylvania	1.6 (0.8 to 2.3)	–2.8 (–3.4 to –2.1)	20	2.0 (1.1 to 2.8)	–3.0 (–3.8 to –2.2)	21
Rhode Island	1.8 (1.0 to 2.5)	–3.5 (–4.3 to –2.7)	21	2.2 (1.3 to 3.0)	–3.4 (–4.2 to –2.5)	22
South Carolina	2.2 (1.3 to 3.2)	–2.9 (–3.8 to –2.0)	20	2.6 (1.6 to 3.7)	–2.9 (–3.9 to –1.9)	21
South Dakota	2.0 (1.3 to 2.7)	–3.5 (–4.3 to –2.7)	21	2.3 (1.5 to 3.1)	–3.0 (–4.0 to –2.1)	21
Tennessee	1.8 (1.0 to 2.6)	–3.1 (–3.9 to –2.3)	22	2.0 (1.1 to 3.0)	–3.1 (–3.9 to –2.2)	22
Texas	2.1 (1.4 to 2.8)	–2.7 (–3.4 to –2.0)	20	2.0 (1.2 to 2.7)	–2.3 (–3.1 to –1.5)	20
Utah	2.0 (1.4 to 2.7)	–3.7 (–4.3 to –3.1)	21	2.2 (1.5 to 3.0)	–3.3 (–4.0 to –2.6)	22
Vermont	1.6 (1.0 to 2.3)	–3.1 (–3.9 to –2.4)	20	1.8 (1.0 to 2.5)	–2.7 (–3.5 to –1.8)	21
Virginia	1.7 (0.9 to 2.4)	–2.9 (–3.7 to –2.2)	21	1.4 (0.5 to 2.3)	–2.3 (–3.1 to –1.4)	22
Washington	1.7 (1.1 to 2.2)	–3.1 (–3.6 to –2.6)	21	2.1 (1.5 to 2.8)	–2.8 (–3.4 to –2.2)	22
West Virginia	1.8 (1.0 to 2.7)	–2.9 (–3.7 to –2.0)	21	2.2 (1.2 to 3.2)	–2.9 (–3.7 to –2.0)	22
Wisconsin	1.7 (0.9 to 2.5)	–3.4 (–4.2 to –2.6)	20	2.2 (1.3 to 3.1)	–3.5 (–4.3 to –2.7)	21
Wyoming	1.8 (1.1 to 2.4)	–3.3 (–4.2 to –2.5)	21	2.1 (1.4 to 2.9)	–3.1 (–4.0 to –2.2)	22

**Abbreviation:** CI = confidence interval.

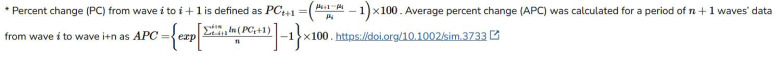

For the same periods that HPS was administered, an association was found between numbers of COVID-19 cases and the frequency of anxiety and depression symptoms. The average number of daily COVID-19 cases was highly positively correlated with anxiety (rho = 0.79) and depression (rho = 0.81) severity scores (Supplementary Table 2, https://stacks.cdc.gov/view/cdc/110123).

## Discussion

The frequency of anxiety and depression symptoms experienced among U.S. adults increased after August 2020 and peaked during December 2020–January 2021. The frequency of symptoms subsequently decreased but in June 2021 remained elevated compared with estimates from the 2019 NHIS. The relative increases and decreases in frequency of reported symptoms of anxiety and depression at both the national and state levels mirrored the national weekly number of new COVID-19 cases during the same period.

An international group of clinicians and mental health experts recommends that during pandemics, delivery systems for mental health care be adapted to mitigate disparities in the provision of health care (*8*). Predicting and planning for fluctuations in demand for behavioral health services is often difficult; however, real-time monitoring of mental health symptoms can provide important information for responding to surges in the demand for mental health services during national emergencies. The observed differences in severity score magnitude and peaks across states in this study indicate that these efforts are important at both the national and state levels.

The findings in this report are subject to at least six limitations. First, modified GAD-2 and PHQ-2 items were administered using a 1-week time frame rather than a 2-week time frame, which might have reduced comparability between HPS and NHIS. Second, short forms were used for GAD and PHQ; restricted ranges of scores might have decreased the likelihood of detecting differences in symptoms nationally or by state. Third, the HPS response rate was <10%, which might have introduced bias; however, trends are less likely than point estimates to be affected, and sample weights somewhat mitigate this bias (*9*). Fourth, peak periods in mental health symptoms overlapped with a break in survey data collection during the holiday season; reported symptoms during that period might have been underestimates if the peak occurred during the break. Fifth, the decrease in the frequency of symptoms observed through June 2021 occurred before the recent surge in COVID-19 cases involving the B.1.617.2 (Delta) variant; that decreasing trend might have slowed or reversed since June. Finally, these data are based on self-report and are subject to recall and social desirability biases.

The increased frequency of reported symptoms of anxiety and depression in this study indicates that mental health services and resources, including telehealth behavioral services, are critical during the COVID-19 pandemic, particularly among populations disproportionately affected by COVID-19. National COVID-19 trends demonstrate that certain populations have been disproportionately affected by high COVID-19 incidence, which also suggests that these populations might be more vulnerable to the psychological consequences of COVID-19. The mental health impact of COVID-19 also might have community-specific effects when morbidity and mortality rates are increasing as a result of COVID-19. Fluctuations in symptoms of anxiety and depression during the pandemic highlight the importance of real-time monitoring of mental health symptoms. Tracking these outcomes, including by demographic characteristics, can provide early indicators of potential increases in the demand for mental health services and for the health care providers needed to treat persons with clinically significant symptoms (*10*).

**Summary**

**What is already known about this topic?**

U.S. Census Bureau Household Pulse Survey data indicate that the percentage of U.S. adults with symptoms of anxiety and depressive disorders increased nationwide from August 2020 to February 2021.

**What is added by this report?**

Nationwide, average anxiety severity scores increased 13% from August to December 2020 and then decreased 26.8% from December 2020 to June 2021. Similar increases and decreases occurred in depression severity scores.

**What are the implications for public health practice?**

Mental health services and resources, including telehealth behavioral services, are critical during the COVID-19 pandemic.

## Footnotes

* U.S. Census Bureau experimental data products are statistical products created using new data sources or previously untested methodologies.

† HPS includes a modified version of the validated PHQ-4 measure for depression and anxiety with a 1-week (versus a 2-week) recall period to reflect the weekly administration of the questions. ­The two anxiety questions asked about 1) feeling nervous, anxious, or on edge and 2) not being able to stop or control worrying. The two depression questions asked about 1) having little interest or pleasure in doing things and 2) feeling down, depressed, or hopeless.

§ Percent change (PC) from wave to is defined as . APC was calculated for a period of waves’ data from wave to wave as .

¶ Data for the 2019 survey year were obtained from NHIS, a survey conducted continuously throughout the year by CDC’s National Center for Health Statistics. NHIS is a nationally representative cross-sectional survey of the U.S. civilian noninstitutionalized population. NHIS uses geographically clustered sampling to select each household; one adult per household is randomly selected to be interviewed. Interviews are generally conducted in respondents’ homes; follow-up interviews to complete surveys might occur by telephone when necessary. Methodological details, protocols, and Ethics Review Board approvals are described elsewhere (https://www.cdc.gov/nchs/nhis/data-questionnaires-documentation.htm). Weighted means for the anxiety and depression severity scores were estimated, accounting for the complex sampling design. Means and CIs for adults aged ≥18 years were estimated using the SAS SurveyMeans procedure.

** 45 C.F.R. part 46.102(l)(2), 21 C.F.R. part 56; 42 U.S.C. Sect.241(d); 5 U.S.C.0 Sect.552a; 44 U.S.C. Sect. 3501 et seq.
